# Genome description of *Phlebia radiata* 79 with comparative genomics analysis on lignocellulose decomposition machinery of phlebioid fungi

**DOI:** 10.1186/s12864-019-5817-8

**Published:** 2019-05-28

**Authors:** Mari Mäkinen, Jaana Kuuskeri, Pia Laine, Olli-Pekka Smolander, Andriy Kovalchuk, Zhen Zeng, Fred O. Asiegbu, Lars Paulin, Petri Auvinen, Taina Lundell

**Affiliations:** 1 Department of Microbiology, Faculty of Agriculture and Forestry, Viikki Campus, University of Helsinki, FI-00014 Helsinki, Finland; 2 DNA Sequencing and Genomics Laboratory, Institute of Biotechnology, Viikki Campus, FI-00014 Helsinki, Finland; 3 Department of Forest Sciences, Faculty of Agriculture and Forestry, University of Helsinki, Viikki Campus, FI-00014 Helsinki, Finland; 4 Present address: VTT Technical Research Centre of Finland Ltd., Espoo, Finland; 5 Present address: Department of Chemistry and Biotechnology, Division of Gene Technology, Tallinn University of Technology, Tallinn, Estonia

**Keywords:** *Phlebia radiata*, comparative genomics, wood decay, carbohydrate-active enzyme genes, lignin biodegradation, co-regulation, peptidases, secondary metabolism, ABC transporters, small secreted proteins

## Abstract

**Background:**

The white rot fungus *Phlebia radiata*, a type species of the genus *Phlebia*, is an efficient decomposer of plant cell wall polysaccharides, modifier of softwood and hardwood lignin, and is able to produce ethanol from various waste lignocellulose substrates. Thus, *P. radiata* is a promising organism for biotechnological applications aiming at sustainable utilization of plant biomass. Here we report the genome sequence of *P. radiata* isolate 79 originally isolated from decayed alder wood in South Finland. To better understand the evolution of wood decay mechanisms in this fungus and the *Polyporales* phlebioid clade, gene content and clustering of genes encoding specific carbohydrate-active enzymes (CAZymes) in seven closely related fungal species was investigated. In addition, other genes encoding proteins reflecting the fungal lifestyle including peptidases, transporters, small secreted proteins and genes involved in secondary metabolism were identified in the genome assembly of *P. radiata*.

**Results:**

The PACBio sequenced nuclear genome of *P. radiata* was assembled to 93 contigs with 72X sequencing coverage and annotated, revealing a dense genome of 40.4 Mbp with approximately 14 082 predicted protein-coding genes. According to functional annotation, the genome harbors 209 glycoside hydrolase, 27 carbohydrate esterase, 8 polysaccharide lyase, and over 70 auxiliary redox enzyme-encoding genes. Comparisons with the genomes of other phlebioid fungi revealed shared and specific properties among the species with seemingly similar saprobic wood-decay lifestyles. Clustering of especially GH10 and AA9 enzyme-encoding genes according to genomic localization was discovered to be conserved among the phlebioid species. In *P. radiata* genome, a rich repertoire of genes involved in the production of secondary metabolites was recognized. In addition, 49 genes encoding predicted ABC proteins were identified in *P. radiata* genome together with 336 genes encoding peptidases, and 430 genes encoding small secreted proteins.

**Conclusions:**

The genome assembly of *P. radiata* contains wide array of carbohydrate polymer attacking CAZyme and oxidoreductase genes in a composition identifiable for phlebioid white rot lifestyle in wood decomposition, and may thus serve as reference for further studies. Comparative genomics also contributed to enlightening fungal decay mechanisms in conversion and cycling of recalcitrant organic carbon in the forest ecosystems.

**Electronic supplementary material:**

The online version of this article (10.1186/s12864-019-5817-8) contains supplementary material, which is available to authorized users.

## Background

Cellulose and hemicellulose are the most abundant natural, renewable carbohydrate polymers in plant cell walls. In the biodegradation and utilization of the plant biomass, it is considered that access to these carbohydrate polysaccharide feedstocks is restricted due to the compact and ordered plant cell wall lignocellulose composite including regions of crystalline cellulose microfibrils, and the aromatic and heterogenous lignin units [1]. Therefore, the use of plant biomass components for different biotechnological applications such as production of bio-based liquid fuels and chemicals requires both physico-chemical and enzymatic treatments to break the composite structure of lignocellulose [2].

In nature, saprotrophic fungi of the *Dikarya* phyla *Ascomycota* and *Basidiomycota* include thousands of species responsible for the important role in decomposition of plant litter and other biomass-based materials [3, 4]. In particular, cycling of carbon and nutrients in the forest ecosystems is largely dependent on fungal decomposition of dead wood, forest litter and soil organic compounds [5, 6]. The fungal phylum *Basidiomycota* contains species with versatile ecological roles and lifestyles such as saprotrophic, plant pathogenic or mutualistic mycorrhizae, and the class *Agaricomycetes* harnesses the most efficient decomposers of wood lignocellulose [7, 8]. Among the *Agaricomycetes*, especially fungi in the phlebioid clade of the order *Polyporales* encompassing corticioid and polyporoid basidiocarp-forming species demonstrate high potential for biotechnological applications due to their ability to attack all chemical components of lignocellulose including lignin [9, 10].

Phylogenetically, the *Polyporales* phlebioid clade fungi may be further divided into genus level sub-clades of *Phlebia*, *Phanerochaete* and *Byssomerulius* [10, 11]. The most studied species with the first published *Basidiomycota* sequenced genome is *Phanerochaete chrysosporium* [12], which is considered as the model white rot fungus for wood decay studies. Since then, draft genomes of several other phlebioid species, also from the genera *Bjerkandera* and *Phlebiopsis*, have been annotated as part of the DOE JGI 1000 Fungal Genomes and related community projects [13–16]. *Phlebia radiata* is a phlebioid white rot fungus of the *Polyporales* family *Meruliaceae* [17] and the type species of genus *Phlebia*. The Finnish isolate *P. radiata* 79 has been widely studied showing the diverse ability to decompose and convert different wood types and plant biomass, to degrade harmful organic compounds, to modify lignin, and to secrete a wide variety of carbohydrate-active enzymes (CAZymes) and oxidoreductases [9, 18, 19]. In particular, the oxidoreductase production properties vital for biological attack on lignins and lignin-like compounds have been of interest [20, 21]. Individual genes encoding class-II lignin peroxidases [22], two divergent class-II manganese peroxidases [23], and two laccases [24, 25] have been cloned and characterized. Transcripts and secreted protein products of these together with an array of CAZyme-encoding genes of *P. radiata* acting against plant cell wall polysaccharides were identified when the fungus was grown on spruce wood [26].

Advances in the development of next-generation genome sequencing platforms have enabled detailed and cost-effective investigation of genomic content of diverse fungi. The *Phlebia* clade includes three genome-sequenced species that are *Phlebia brevispora* [7], *Phlebia centrifuga* [27], and *Phlebia radiata* presented in this study. Among the *Phanerochaete* clade, genomes of *Phanerochaete chrysosporium* [28], *Phanerochaete carnosa* [29], *Bjerkandera adusta* [7] and *Phlebiopsis gigantea* [15] have been assembled and annotated. The number of genome-sequenced fungi representing different lifestyles has been accumulating particularly within the 1000 Fungal Genomes project [13, 16, 28]. The gene content and genomic arrangement apparently reflect the lifestyle of an organism; in fungi, comparative genomics approaches have given explanations for e.g. brown rot versus white rot decay of wood [8, 13, 30], and divergent and specific mycorrhizal associations with tree and other plant roots [31]. With the aid of whole-genome sequencing together with comparative genomics, genes encoding enzymes and proteins vital for the organism in its natural habitat, evolutionary relationships between fungal species and clades may as well be predicted [8, 13, 14, 28–31]. Comparative genomics studies may also enable identification of novel activities important for plant biomass degradation and deepen the overall understanding of the fungal wood decay genetics and mechanisms.

In this study, high-throughput genome characterization of *P. radiata* as well as comparative analysis on genes associated with the plant cell wall degradation among the phlebioid fungal species (altogether seven species genomes) were elucidated. Special attention was given to the CAZyme-encoding genes involved in cellulose, hemicellulose and pectin degradation, and in oxidative attack on lignin. Other genes reflecting the lifestyle of the fungus including peptidase, ABC transporter, small secreted protein (SSP) and genes involved in secondary metabolism were also identified in the genome and discussed.

## Results

### Characteristics of the *Phlebia radiata* genome assembly

In total, the genome assembly contained 93 contigs as unitigs at the average of 72X sequencing coverage. The assembly included the mitochondrial mtDNA [82] in a single contig which was further removed, resulting in final assembly of 40.4 Mbp (haploid genome size) containing 92 nuclear contigs as scaffolds (Table 1). Mean read length was 5 552 bp, average contig size 436 607 bp, maximal contig size 3 403 443 bp, minimal contig size 5 203 bp and GC content 53 %. In total, 14 082 unique gene models and 547 possible splice variants were recognized by the aid of available RNA sequencing data using the *ab initio* gene prediction software BRAKER1.

**Table 1:** Summary of the genome assembly features of the phlebioid fungal genomes included in the comparative study

Genome features	*P. radiata*	*P. brevispora*	*P. centrifuga*	*B. adusta*	*P. gigantea*	*P. carnosa*	*P. chrysosporium*
Assembly size (Mbp)	40.41	49.96	34.85	42.73	30.14	46.29	35.15
# of contigs	92	3178	3667	1263	1195	2272	1253
# of scaffolds	92	1645	3022	508	573	1137	232
# of scaffolds ≥ 2 Kbp	92	1645	1652	508	506	1136	204
Scaffold L50	8	12	201	13	72	6	8
Scaffold N50 (Mbp)	1.98	1.31	0.05	1.03	0.12	3.53	1.91
# of gaps	0	1533	645	755	622	1135	1021
Average gene lenght (bp)	1847	1627	1378	1703	1714	1765	1684
Average transcript length (bp)	1463	1274	1134	1388	1380	1446	1401
Average exon length (bp)	227	225	274	248	230	269	259
Average intron length (bp)	72	78	80	71	69	75	66
Average protein length (bp)	488	400	378	406	411	384	408
Exons per gene	6.46	5.66	4.14	5.59	6	5.36	5.41
# of gene models	14629	16170	13785	15473	11891	13937	13602

Data were collected from the JGI MycoCosm (genome.jgi.doe.gov/programs/fungi/index.jsf)

*P. radiata* genome assembly was compared with other phlebioid clade fungal genomes [10, 11] including the species *Phlebiopsis gigantea*, *Phlebia brevispora*, *Phlebia centrifuga*, *Bjerkandera adusta*, *Phanerochaete chrysosporium* and *Phanerochaete carnosa* (Table 1). Genomic data of all the species are available at the Joint Genome Institute (JGI) MycoCosm (genome.jgi.doe.gov/programs/fungi/index.jsf) [16]. Noticeable is the quality of *P. radiata* genome with 0 gaps in the closed and gene annotated assembly, and with contigs extended to <100 scaffolds (Table 1). Scaffold L50 value 1.98 Mbp is near to estimated median average chromosomal length (between 0.8 – 3.5 Mbp). The species *P. gigantea*, *P. centrifuga* and *P. chrysosporium* had somewhat smaller genome sizes (30-35 Mbp) than the other phlebioid species. The average length of *P. radiata* gene and transcript models were the largest among the studied fungi (1847 bp and 1463 bp, respectively). Noticeable is also the highest number of recognized intron-exon junctions in the gene models of *P. radiata* (average exon number 6.46 per gene; Table 1).

### Functional annotation of the *P. radiata* gene models

**Fig. 1:**
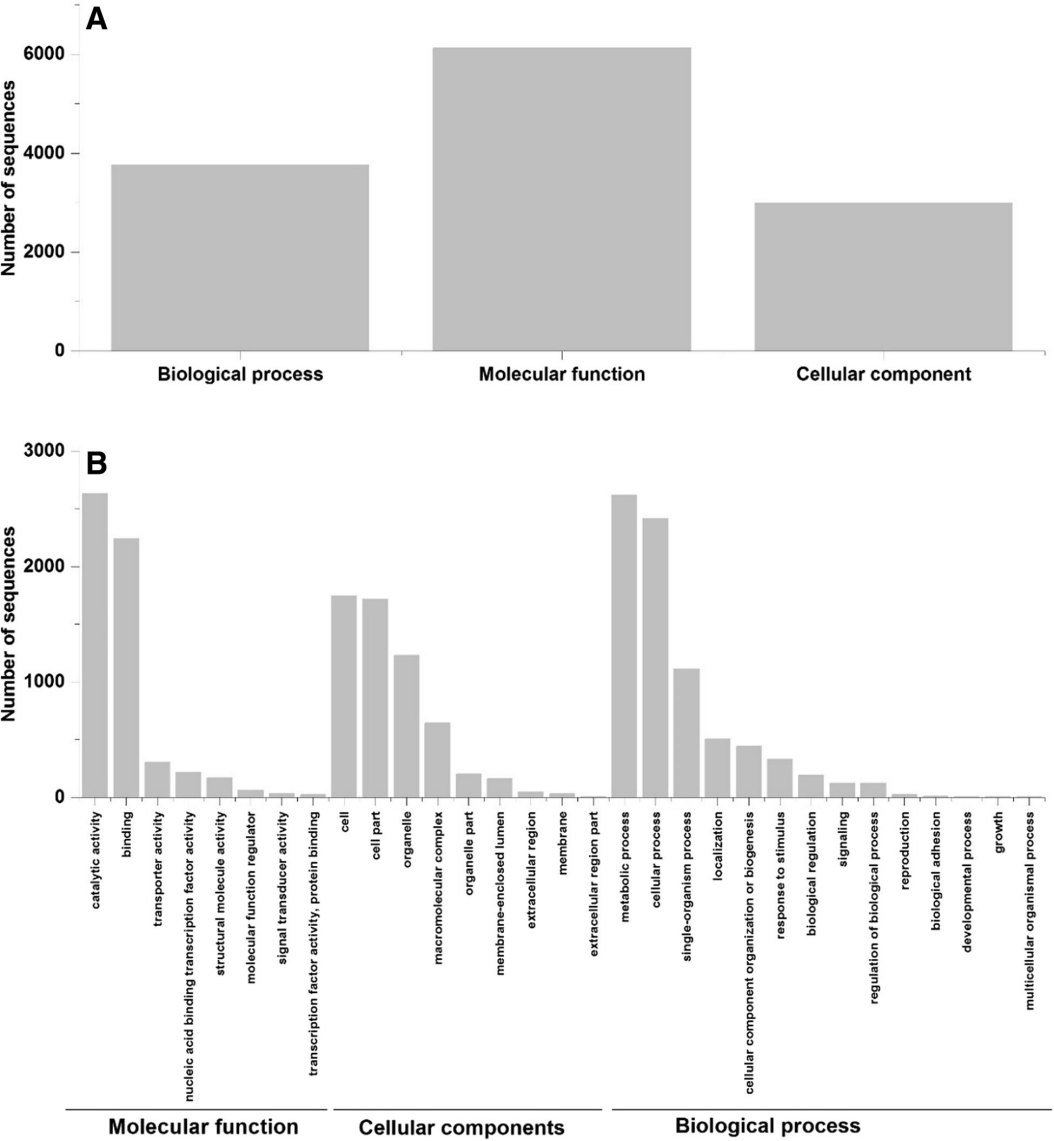
Gene Ontology classification of *P. radiata* proteins. Functional classification by GO categories (**a**) and GO terms (**b**)

The presence of 5´ upstream open reading frames (uORFs), high number of introns and very short exons (from a few to even less than one codon in some cases) were found to be typical for *P. radiata* genes. For translated 365 gene models, no blastp or protein domain hits were obtained. According to Gene Ontology classifications (Fig. 1a, b), the majority of *P. radiata* predicted proteins were assigned to the functional terms of “Catalytic activity”, “Binding”, “Metabolic process” and “Cellular process” (Fig. 1b).

### GO enrichment analysis

GO enrichment analysis was conducted based on the previous RNA-seq transcriptome data of *P. radiata* cultivated both on solid spruce wood (2-week and 4-week time points) and for comparison, on liquid malt extract medium (2-week time point) [26]. GO terms over-represented among the genes which were up-regulated at both time points on spruce wood included the terms of “carbohydrate metabolic process”, “cell wall”, “oxidoreductase activity”, “hydrolase activity, acting on glycosyl bonds”, “extracellular region”, “external encapsulating structure”, “catalytic activity”, “cell wall organization or biogenesis” and “lipid metabolic process” (Additional file 1). The enriched term of “carbohydrate metabolic process” was statistically the most significant (P-value 1.03 x 10^-19^). At the four week time point on spruce wood substrate, the most enriched GO terms among the up-regulated genes included the “transmembrane transport” and “establishment of protein localization to membrane”. Much greater number of GO terms were enriched only among the genes which were up-regulated at the 2-week time point on spruce wood. These included for example GO terms referring to organic acid metabolism (“carboxylic acid metabolic process”, “organic acid metabolic process”, “oxoacid metabolic process”) and to translation (“structural constituent of ribosome”, “ribosome”, “translation”, “ribonucleoprotein complex biogenesis”, “intracellular ribonucleoprotein complex”, “ribosome biogenesis”, “ribonucleoprotein complex”).

### Carbohydrate-active enzyme genes

Carbohydrate-active enzyme encoding genes of *P. radiata* were annotated previously during the combinatory transcriptome and proteome study on wood [26]. Consequently, a comparative analysis on the number of CAZyme-encoding genes of *P. radiata* and six other sequenced phlebioid species of *Polyporales* fungi (Table 2) was performed.

**Table 2:** Number of the identified CAZyme-encoding genes involved in breakdown of plant cell wall lignocellulose components in the genomes of the studied phlebioid fungi

*Cellulose active*	*P. radiata*	*P. brevispora*	*P. centrifuga*	*B. adusta*	*P. gigantea*	*P. carnosa*	*P. chrysosporium*
GH1	2	2	2	2	2	2	2
GH3	10	8	7	9	9	11	10
GH5_5	4	4	2	4	4	6	2
GH5_22	2	2	2	2	2	2	2
GH6	1	1	1	1	1	1	1
GH7	6	4	3	5	5	5	8
GH9	1	1	1	1	1	1	1
GH12	2	2	2	2	3	3	2
GH44	1	2	1	0	0	0	0
GH45	3	3	1	1	1	1	2
GH131	2	4	2	3	2	2	3
*Hemicellulose active*							
CE1	2	1	1	1	2	3	4
CE5	0	1	0	0	0	0	0
CE15	2	2	6	2	1	3	2
CE16	10	8	6	15	6	5	7
GH2	3	2	3	3	3	2	2
GH5_7	3	2	3	2	2	2	3
GH10	7	8	6	4	4	5	6
GH11	1	0	0	0	2	1	1
GH27	2	2	2	3	3	3	3
GH29	1	1	1	0	0	0	0
GH31	4	5	6	4	6	8	6
GH35	3	4	3	4	2	4	3
GH43	2	2	2	6	7	4	4
GH51	1	1	4	2	2	2	2
GH74	2	1	1	2	2	2	4
GH95	1	3	1	1	0	1	1
GH115	1	2	2	2	1	1	1
*Pectin active*							
CE8	2	3	1	2	4	2	2
CE12	0	0	0	1	1	0	0
GH28	7	5	5	6	10	4	5
GH53	1	1	1	1	1	1	1
GH78	2	1	2	2	1	1	1
GH88	1	1	1	1	1	1	1
GH105	3	0	2	1	0	0	0
PL1	0	0	0	1	0	0	0
PL4	1	1	1	1	0	0	0
*Auxiliary redox enzymes*							
AA1	7	11	6	2	5	10	5
AA2	10	15	8	21	9	11	15
AA3	32	39	23	39	23	36	38
AA3_1 (CDH)	1	1	1	1	1	1	1
AA5	10	8	6	7	6	6	7
AA9	12	12	11	28	15	11	16
AA14	3	2	2	3	3	2	2
DyP	1	3	2	10	4	1	0
HTP	5	2	4	4	4	3	3

CAZy GH, CE, PL and AA classes, dye-decolorizing peroxidases (DyP), and heme-thiolate peroxidases (HTP) are indicated

In total, 209 glycoside hydrolase (GH), 27 carbohydrate esterase (CE) and 8 polysaccharide lyase (PL) genes were identified in the *P. radiata* genome (Additional file 2). Of these genes, 76 GHs, 15 CEs and 1 PL were annotated to putatively encode enzymes involved in breakdown of plant cell wall lignocellulose. In addition, twelve auxiliary activity family 9 (AA9) lytic polysaccharide monooxygenase (LPMO) encoding genes and three genes coding for AA14 lytic xylan oxidases were recognized in the genome. The carbohydrate-binding module (CBM) for cellulose-binding domain (IPR000254) was detected in 32 genes coding for AA9 LPMOs, and exoglucanases of the families GH6, GH7 and GH131, one GH3 β-glucosidase, endoglucanases of the families GH5 and GH45, xylanases of the families GH10 and GH11, GH5 mannanases, one CE1 acetyl xylan esterase, one CE15 glucuronoyl esterase, and one CE16 acetylesterase (Additional file 2). In addition, four CBM1 domain-containing proteins with no homology to CAZyme activity catalytic domains were detected in *P. radiata*. One of these genes encodes a putative carbohydrate-binding iron reductase whereas another protein with CBM1 demonstrated homology to phosphodiesterases.

### Cellulose breakdown

**Fig. 2:**
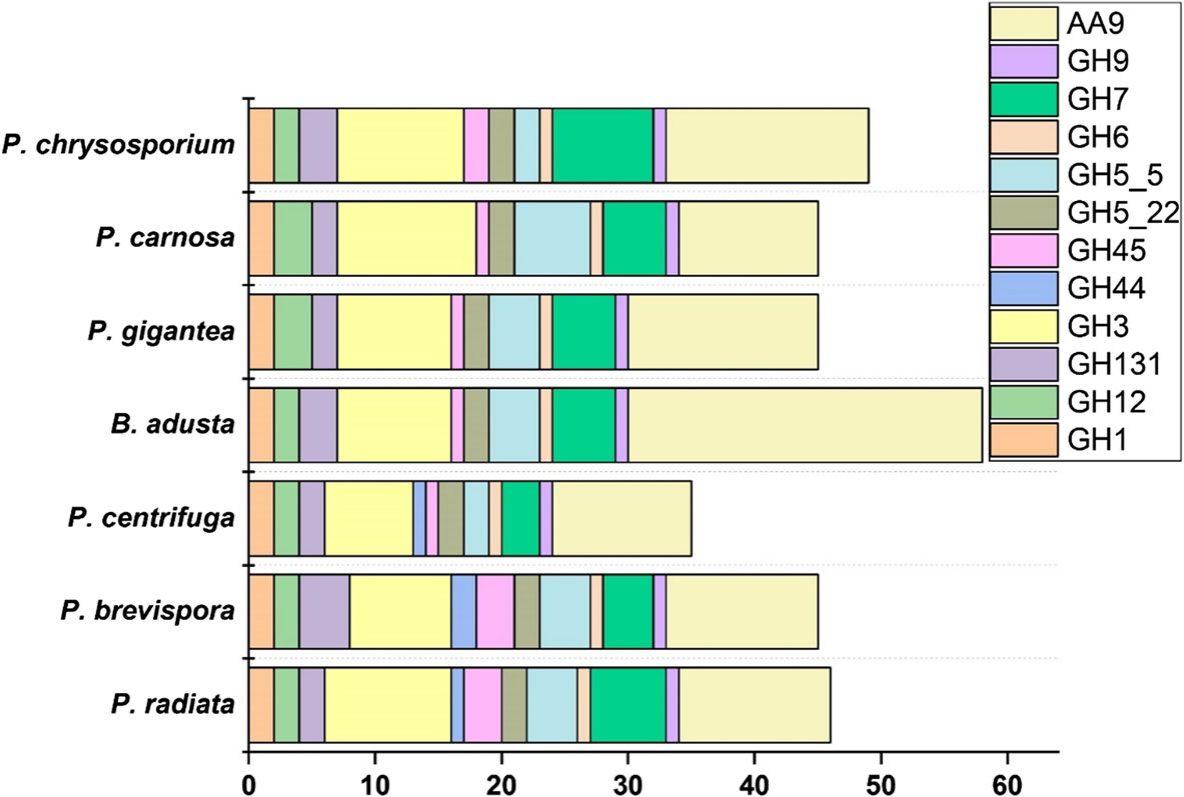
Number of cellulose decomposing-enzyme coding genes in the genomes of the phlebioid fungi

As could be expected, all the studied phlebioid genomes possess genes encoding activities for the hydrolytic decomposition of polymeric cellulose including cellobiohydrolases, endoglucanases and β-glucosidases (Fig. 2). Largest number of cellulolytic genes was found in the CAZy families GH3 and GH7 as well as in the auxiliary oxidoreductase family AA9 (Fig. 2). Typical for *Polyporales* white rot fungi, one gene encoding GH6 (non-reducing end active cellobiohydrolase) was conserved in all phlebioid genomes whereas the number of genes coding for GH7 (reducing end active cellobiohydrolases) ranged from three up to eight (Table 2). *P. radiata* genome harbors six genes coding for GH7 proteins, of which three are unique and three have shared protein homology, indicating sequential gene duplications. The gene models minus.g2003 and plus.g2026 contain only 10 and 14 nt (nucleotide) differences, respectively. The third gene model minus.g8589 contains two 44 nt differences and 2 nt deletions resulting in e.g. variation in translation for two codons in comparison to the other two (together identical) protein models. The single nucleotide polymorphism (SNP) detected among the duplicates occurs at the codon third position. Variations in the GH7 proteins of *P. radiata* and the other phlebioid fungi are frequent in the protein model linker region separating the catalytic domain and fused CBM1 domain.

CAZy family GH5 comprises hydrolytic activities on various polysaccharide substrates present in the plant cell wall and in the fungal cell wall [32]. Hence, GH5 sub families 5, 7 and 22 were included in this comparative study. Family 5_5 includes endo-β-1,4-glucanase (EC 3.2.1.4) activities, family 5_7 endo-β-1,4-mannanase activities (EC 3.2.1.78) and family 5_22 activities of endo-beta-1,4-glucanase and β-xylosidase. Of the seven phlebioid genomes, *P. centrifuga* and *P. chrysosporium* possess only two GH5_5 endo-β-1,4-glucanase encoding genes whereas the other genomes contain at least four genes (Table 2). Interestingly, the family GH44 endoglucanases were identified as one or two gene copies only in the three *Phlebia* species. *P. radiata* and *P. brevispora* have three genes encoding GH45 endoglucanase, whereas the other phlebioid fungi contained less, one or two genes. Noteworthy is that all phlebioid genomes possess one copy of a putative GH9 cellulase encoding gene.

All phlebioid fungal genomes apparently harbor two GH1 β-glucosidase-encoding genes, whereas seven or more GH3 family β-glucosidase-encoding genes were identified (eight genes in *P. radiata*) (Table 2, Fig. 2). CAZy family GH3 is a complex family including also activities affecting the fungal cell wall [33]. Two to three GH12 family genes encoding putative endoglucanases and xyloglucan-specific endo- β-1,4-glucanases were equally present in the phlebioid fungi. In addition to the traditional endoglucanase and cellobiohydrolase-encoding genes, the phlebioid genomes contain 2-4 putative members of the family GH131 broad specificity β-glucanases. One of the two *P. radiata* GH131 protein models included a C-terminal CBM1 domain indicating possible attachment to polymeric cellulose. In addition to the hydrolytic cellulolytic enzymes, all phlebioid genomes harbor a large number (11-28) of AA9 lytic polysaccharide monooxygenase encoding genes (Fig. 2) involved in oxidative decomposition of cellulose [34].

### Hemicellulose degradation

**Fig. 3:**
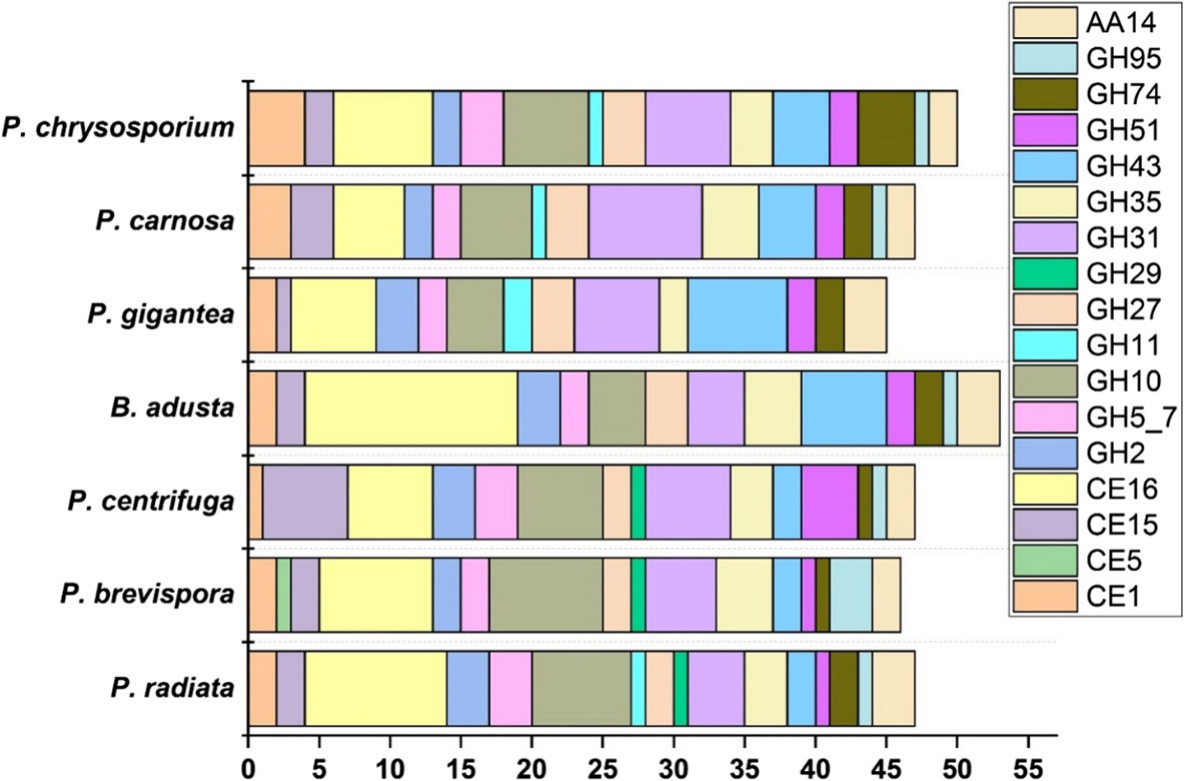
Number of genes encoding hemicellulose-decomposing enzymes in the genomes of the phlebioid fungi

Genomes of the phlebioid fungi possess a wide array of genes encoding all the necessary activities for the decomposition of glycosidic and ester linkages of diverse hemicelluloses (Fig. 3). Largest number of genes were detectable in the carbohydrate esterase family CE16 (acetylesterase activity) ranging from 5 to 15 genes per genome (Table 2), and glycoside hydrolase families GH10, GH31 and GH43 (Fig. 3). However, the GH29 α-L-fucosidase encoding genes appear to be specific for the three *Phlebia* species (Table 2) whereas the GH11 endoxylanase encoding genes were absent in the genomes of *P. brevispora*, *P. centrifuga* and *B. adusta*. Interestingly, *P. gigantea* genome harbors two genes for GH11 endoxylanase while missing the GH95 α-fucosidase activity genes. A putative CE5 acetyl xylan esterase gene was detected only for *P. brevispora*.

Genes encoding the novel AA14 oxidoreductase family of lytic xylan oxidases/monooxygenases were identified in *P. brevispora*, *P. gigantea*, *B. adusta*, *P. chrysosporium* and *P. carnosa* in a previous study [35]. For *P. radiata*, three putative genes with translated amino acid sequence homology to the *Pycnoporus coccineus* AA14 proteins were identified by blastp search of the genome protein models (Table 2). For *P. centrifuga*, two possible AA14 homologs were found by blastp search against the MycoCosm genome data. Interestingly, two of the *P. radiata* AA14 genes encode proteins with an extension in the C-terminus (3´ end) whereas the third gene protein model is shorter in the C-terminus than the corresponding *P. coccineus* AA14 homologs. All three *P. radiata* AA14 protein models demonstrate a conserved N-terminal histidine after the signal sequence cleavage site, similar to the features of the *P. coccineus* proteins [35]. Whereas one of the AA14 genes was highly up-regulated in *P. coccineus* cultivated on pine and aspen substrates [36], the *P. radiata* homologs were more constitutively expressed on spruce wood [26] (Additional file 2).

### Pectin degradation

**Fig. 4:**
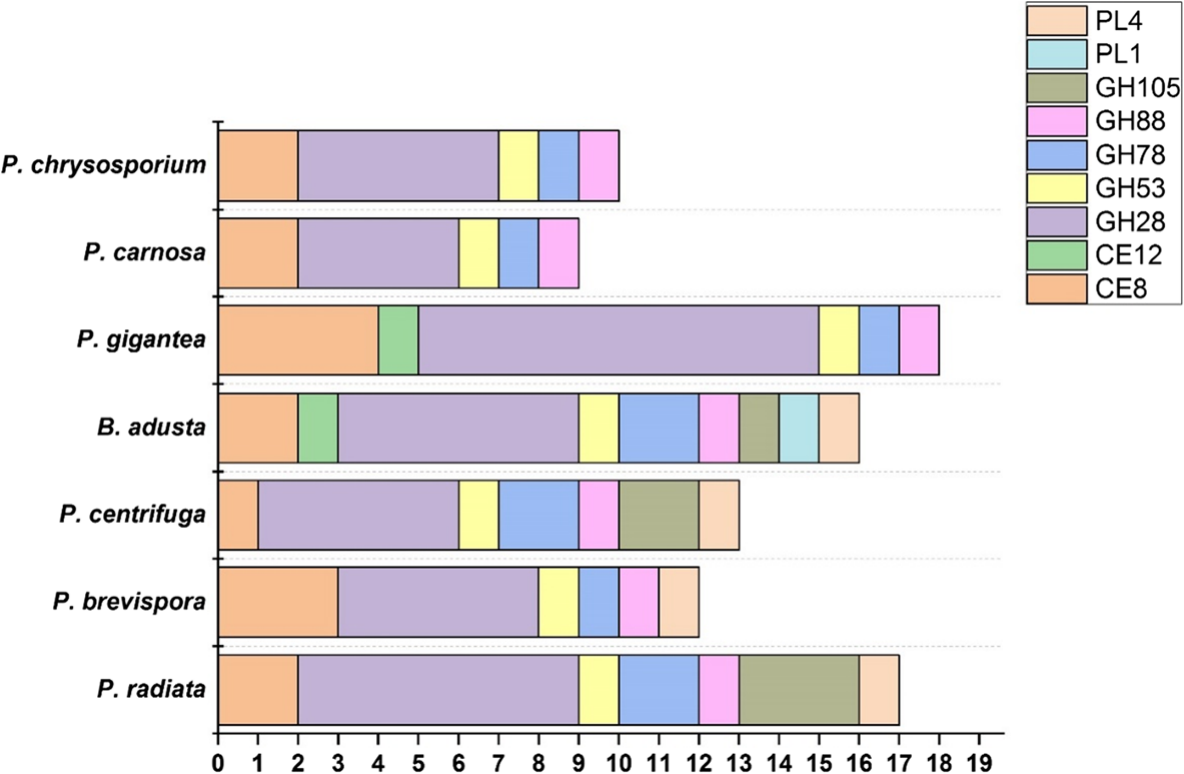
Number of pectin-decomposing enzyme coding genes in the genomes of the phlebioid fungi

From the CAZy families including pectin-decomposing activities, GH28, GH105 and CE8 families had the largest number of genes (Fig. 4). The number of family GH28 polygalacturonase and rhamnosidase encoding genes was relatively large (4-10 genes) in the phlebioid genomes, especially in the *P. gigantea* genome (Table 2). Also, four genes encoding CE8 pectin methylesterases were identified in *P. gigantea* genome. Differences were noticed in the CE12 family including acetylesterase activity against pectin with genes detected only in *B. adusta* and *P. gigantea*, and family GH105 rhamnogalacturonyl and glucuronyl hydrolase activity encoding genes which were identified (1-3 genes) only in the genomes of *P. radiata*, *P. centrifuga* and *B. adusta*. Of the potential pectin-acting lyases, one gene encoding family PL4 rhamnogalacturonan endolyase activity was restricted to the three *Phlebia* species and *B. adusta*, whereas family PL1 pectin/pectate lyase (one gene) was specific for *B. adusta* (Fig. 4, Table 2).

### Lignin conversion

**Fig. 5:**
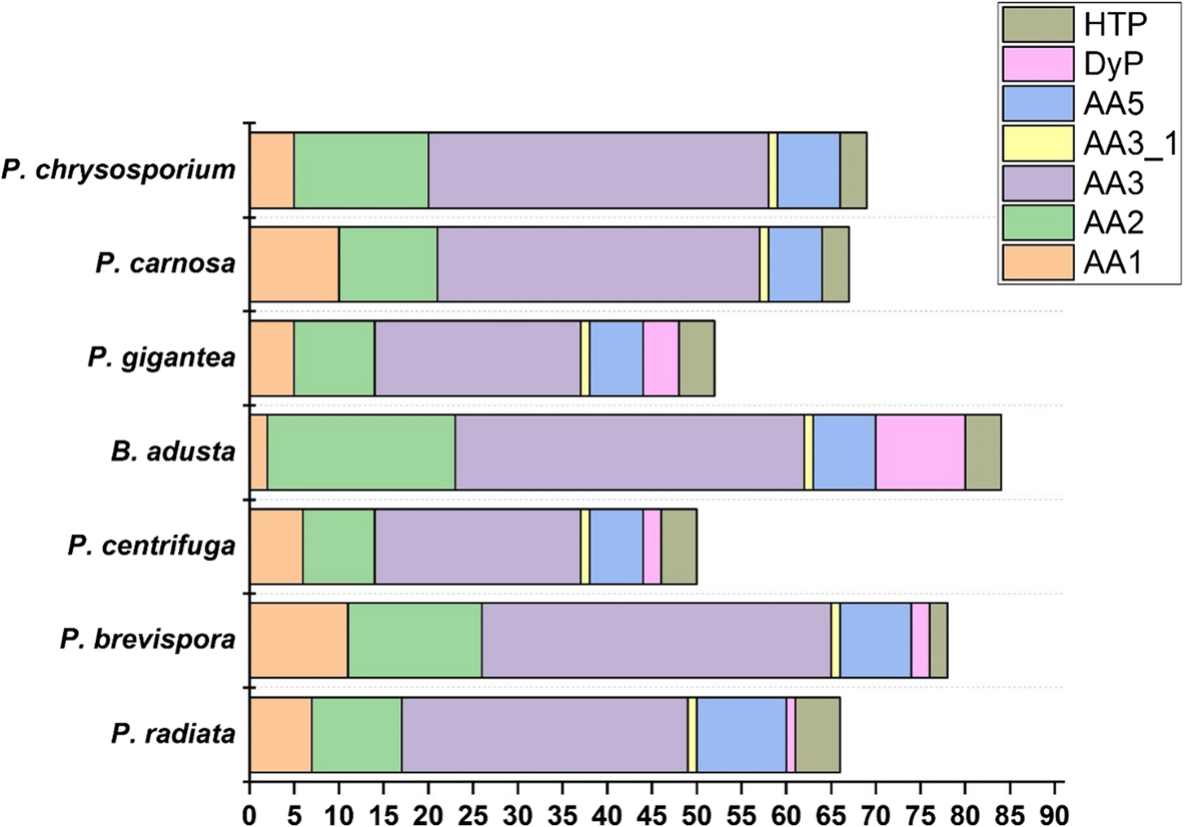
Number of lignin-modifying and auxiliary oxidoreductase-coding genes in the genomes of the phlebioid fungi

In total, 65 CAZy auxiliary activity (AA) class genes encoding lignin-modifying and associated redox activities were identified in *P. radiata* genome (Additional file 2). All seven phlebioid species harness multiple AA1 laccases/ferroxidases/laccase-like multicopper oxidases and multiple AA2 family class-II heme-including, lignin and manganese peroxidases (LiP, MnP) (Table 2, Fig. 5). Notably, the three *Phlebia* species include several genes encoding both long-MnP and short-MnP AA2 peroxidases [14, 23] together with lignin peroxidase encoding genes, whereas *B. adusta* genome encodes one additional class-II versatile peroxidase (VP) [13, 14]. Auxiliary enzymes possibly involved in providing hydrogen peroxide for the lignin-modifying peroxidases include, for example, family AA3 glucose-methanol-choline (GMC) family oxidases (glucose/aryl alcohol/alcohol/pyranose oxidases) and AA5 copper radical oxidases (galactose/glyoxal oxidases). Members of these gene families were present in the phlebioid genomes. Importantly, one copy of the cellulolytic and lignin-attacking oxidative activity interlinking AA3_1 oxidoreductase family cellobiose dehydrogenase (CDH) encoding gene is present in all of the phlebioid genomes. In addition, *P. radiata* genome includes one gene encoding a putative dye-decolorizing peroxidase (DyP) and five genes encoding putative chloroperoxidase-like heme-thiolate peroxidases (HTP) [37]. Presence of a few putative HTPs and 1-4 DyPs is a common feature among the phlebioid fungi (Table 2), thereby extending their functional potential in oxidation and activation of lignin-like and various phenolic compounds [37]. Exceptional is *B. adusta* with 10 potential DyP encoding genes recognized in the genome, and the absence of DyP genes in *P. chrysosporium* (Table 2, Fig. 5).

### Genomic clustering of CAZyme genes

Carbohydrate-active enzyme encoding genes under consorted expression and regulation mechanisms were searched from the *P. radiata* genome assembly according to the criteria of (i) close genomic coordinates, and (ii) up-regulation of gene expression on spruce wood as growth substrate in our pervious study [26] (Additional file 3). The most interesting clusters include genes encoding activities against plant cell wall components and genes showing strong co-regulation. *P. radiata* CAZyme genes were identified and annotated in 33 unitigs whereas the majority (54 %) of the CAZyme genes were assigned to seven distinct unitigs. When applying the sliding window approach [38], 55 CAZyme genes were assigned to clusters. Minimum of three CAZyme genes in a window of maximum of 11 genes was considered as a CAZyme cluster. The size of the cluster window was calculated by dividing the number of *P. radiata* gene models (14082) with the number of annotated CAZyme genes (244). Hence, when random distribution of CAZyme genes was expected, one CAZyme gene should appear once within 58 subsequently ordered genes. When the three CAZyme genes of the cluster are expected to be separated from each other by less than a fifth of the average distribution number, the size of the window was 11 (0.2 x 58). A total of 17 clusters were detected according to these criteria (Additional file 3). In addition, interesting clusters including two CAZyme genes next to each other and encoding plant cell wall degrading activities are also included in the data set (Additional file 3).

**Fig. 6:**
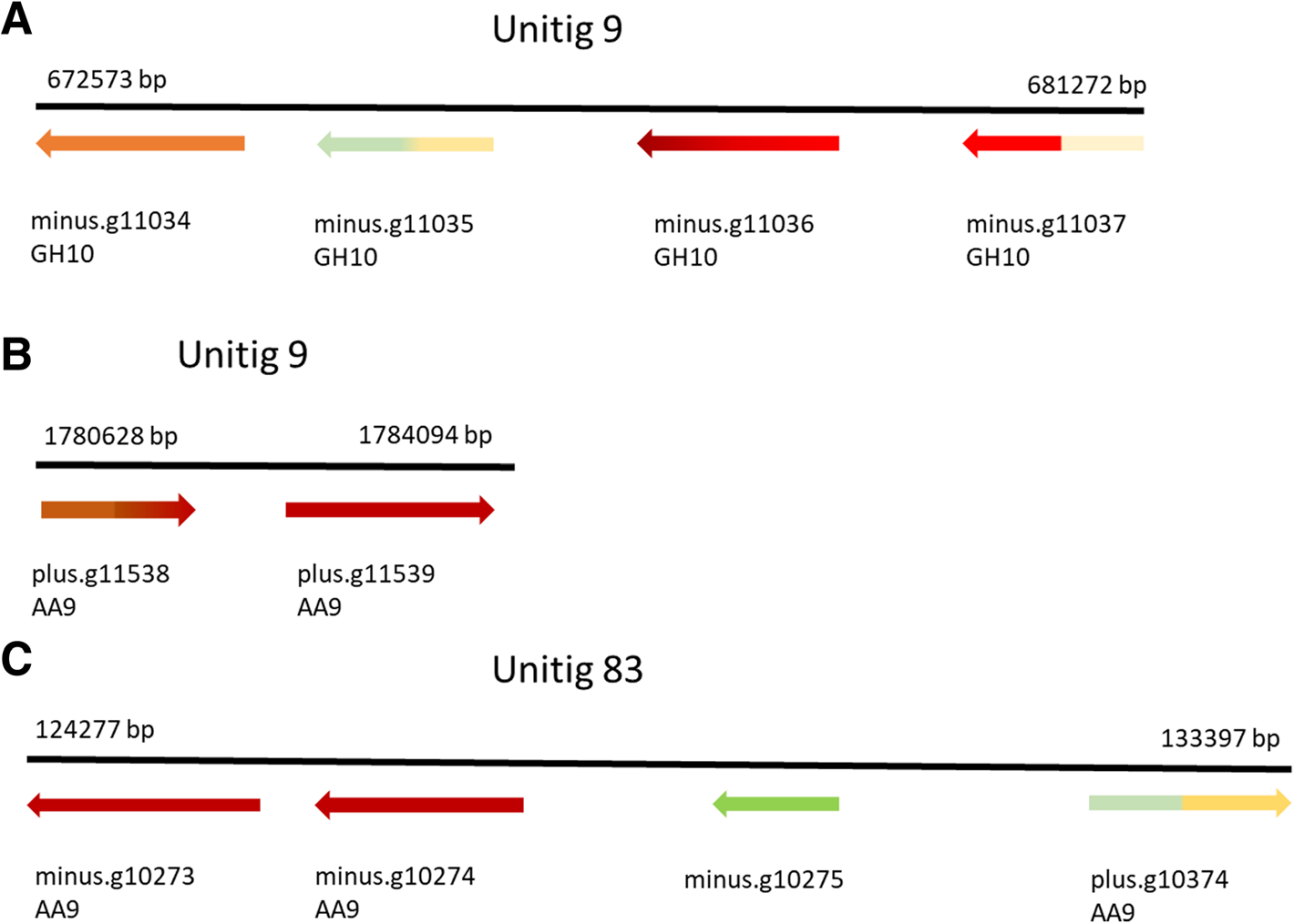
Clustering and co-regulation of GH10 endo-1,4-β-xylanase (**a**) and AA9 LPMO encoding genes (**b**, **c**) of *P. radiata*. Intensity of the color indicates the scale of up- or down-regulation (dark red for high up-regulation, green for down-regulation, yellow for no significant difference) of gene expression on spruce wood substrate [26]. Direction of the arrow indicates gene location and orientation (on plus or minus strand) in the unitigs of the genome assembly (this study). The arrowhead indicates the 2-week time point of spruce wood cultivation whereas the end of the arrow refers to the 4-week time point. Beginning and end of the cluster (as genomic coordinates) is marked above the unitig. Gene model minus.g10275 in unitig 83 encodes a non-CAZyme protein

In general, no clear tendency for tight clustering according to genomic localization could be asserted to highly similar expression patterns of the multiple genes encoding diverse CAZy classes and families. However, clustering, and in some cases, concerted expression and possible co-regulation of the members of GH10 (xylanase activity) and AA9 (LPMO) family genes was detected (Figure 6). There is a cluster of four putative GH10 endo-beta-1,4-xylanase encoding genes in near location and antisense orientation in the unitig 9 of *P. radiata* genome assembly (Fig. 6a). Three of the genes were up-regulated on spruce wood substrate. In unitig 9, two AA9 lytic polysaccharide monooxygenase encoding genes are neighbours and strongly up-regulated at both time points on spruce wood substrate (Fig. 6b). In unitig 83, three AA9 genes and a non-CAZyme protein encoding gene (minus.g10275) form a cluster (Fig. 6c). From the three AA9 genes, two are strongly up-regulated on spruce whereas the third AA9 gene was up-regulated only at the two-week time point of spruce cultivation. In unitig 6, another set of additional two AA9 genes are located next to each other but only one of the genes was up-regulated on spruce wood (Additional file 3).

Clustering of GH10 enzyme encoding genes was similarly recognized to be shared by the three species of the genus *Phlebia* (*P. radiata*, *P. brevispora*, *P. centrifuga*) but not in the three other phlebioid fungi studied (Table 3). Clustering of 2-6 GH10 genes is interrupted in the *Phlebia* genomes by a maximum of three non-CAZyme protein encoding genes. However, considering AA9 LPMO encoding genes, majority of the phlebioid species demonstrated several AA9 gene clusters in different scaffolds (2-4 clusters) except for the *P. centrifuga* showing only one cluster of two AA9 encoding genes (Table 4).

**Table 3:** Genomic clusters of GH10 endoxylanase encoding genes in the genome assemblies of the phlebioid fungi

Fungus	Cluster location in the genome assembly	Number of genes in the cluster	Number of non-CAZyme genes in the cluster
*P. radiata*	Unitig 9/Scaffold 8:672573-681272	4	0
*P. brevispora*	Scaffold 12:834597-856661	9	3
*P. centrifuga*	Scaffold 2925:6494-13943	3	0
*B. adusta*	absent	-	-
*P. gigantea*	absent	-	-
*P. carnosa*	Scaffold 3:3347462-3351815	3	1
*P. chrysosporium*	absent	-	-

Locations of *P. radiata* gene models are indicated as original assembly unitig positions, and as scaffold positions of the MycoCosm genome repository. Other data were retrieved from the MycoCosm

**Table 4:** Genomic clusters of AA9 encoding genes in the genome assemblies of the seven phlebioid fungi

Fungus	Cluster location in the genome assembly	Number of genes in the cluster	Number of non-CAZyme genes in the cluster
*P. radiata*	Unitig 6/Scaffold 23:237827-242972	2	0
	Unitig 9/Scaffold 8:1780628-1784094	2	0
	Unitig 83/Scaffold 30:124277-133397	4	1
*P. brevispora*	Scaffold 7:1939036-1942768	2	0
	Scaffold 8:1713073-1722627	4	1
	Scaffold 9:703327-712085	3	1
*P. centrifuga*	Scaffold 809:16725-20452	2	0
*B. adusta*	Scaffold 3:699996-705149	3	0
	Scaffold 12:565705-571395	3	0
	Scaffold 19:267134-284710	9	2
	Scaffold 35:28836-31774	2	0
*P. gigantea*	Scaffold 13:72697-82083	4	2
	Scaffold 167:19857-23789	2	0
	Scaffold 240:27459-32596	3	0
*P. carnosa*	Scaffold 3:465671-473281	3	0
	Scaffold 10:958421-970775	4	2
*P. chrysosporium*	Scaffold 7:1587181-1592893	4	1
	Scaffold 10:522449-536923	7	4

Locations of *P. radiata* gene models are indicated as original assembly unitig positions, and as scaffold positions of the MycoCosm genome repository. Other data were retrieved from the MycoCosm

Other CAZyme gene clusters of interest and identified in *P. radiata* genome included, for example, two GH45 endoglucanase encoding genes located next to each other in unitig 0 while only one of the genes was strongly up-regulated on spruce wood (Additional file 3). Of three CE16 carbohydrate esterase encoding genes forming a cluster in the same unitig, two were up-regulated on spruce wood whereas one gene was down-regulated. Two up-regulated GH35 genes were identified next to each other in unitig 23. In addition, two GH3 β-glucosidase encoding genes located next to each other in unitig 59 and two GH28 encoding genes next to each other in unitig 96, but these genes were not co-regulated (one gene was up-regulated while the other was down-regulated) thereby indicating individual regulation of their expression. On the contrary, two family GH5 annotated genes were found adjacently located in unitig 117 and convergently expressed - up-regulated at both time points on the spruce wood substrate. Moreover, both of the encoded proteins demonstrated high homology with a specific *P. chrysosporium* β-xylosidase that cleaves xylan hemicellulose chains synergistically with endo-xylanases [39].

Of the ten identified AA2 class-II peroxidase encoding genes of *P. radiata* targeted to lignin modification (Table 2), *lip3* and *lip4* genes are closely located in the genome, and separated only by one gene encoding a hypothetical protein in unitiq 9. Surprisingly, the genes were not co-regulated on spruce wood substrate [26]. Similarly, the AA1 laccase encoding *lacc1* and *lacc2* genes of *P. radiata* located in unitig 55 are neighbours and possess similar expression profiles but, however, were not strongly up-regulated on spruce wood [26]. In unitig 70, one AA8 iron reductase encoding gene is seemingly co-expressed with a GH128 β-glucanase encoding gene. Interestingly, the only cellobiose dehydrogenase (CDH) encoding gene of *P. radiata* was recognized to situate next to a putative catalase encoding gene in the same unitig 70, with concurrent up-regulation at both time points on spruce wood. Of the potential hydrogen peroxide producing extracellular enzymes, three AA5 copper radical oxidase encoding genes were identified in unitig 87 as separated by two intervening non-CAZyme genes. Two of these AA5 genes were up-regulated on spruce, whereas the third gene in the middle of the cluster was down-regulated. The above described clustering of functionally related or similar genes suggest that despite their close structural location in the genome, genes in the same cluster may display individual and deviated pattern of regulation of expression.

### Peptidases

Annotation of the putative *P. radiata* peptidases according to the classifications in the MEROPS database [40] (https://www.ebi.ac.uk/merops/) indicated that at least 327 genes identified in the *P. radiata* genome possibly encode proteolytic enzymes (Table 5). Highest number of the genes were classified to code for serine peptidases. Among the seven phlebioid species, *P. radiata* was the only species of the genus *Phebia* comprising genes (2 genes) encoding putative glutamic peptidases. However, more than ten genes encoding glutamic peptidases were detected in the two species of *Phanerochaete*, and in *P. gigantea*.

**Table 5:** Number of peptidase encoding genes identified in the genomes of the seven phlebioid fungi

Peptidase family Fungal species	Aspartic	Cysteine	Glutamic	Metallo	Serine	Threonine	Sum of peptidases
*P. radiata*	48	50	2	60	149	18	327
*P. brevispora*	67	59	0	73	207	19	425
*P. centrifuga*	58	45	0	51	138	18	310
*B. adusta*	60	69	0	68	173	18	388
*P. gigantea*	43	51	15	62	159	18	348
*P. carnosa*	70	91	34	61	170	17	443
*P. chrysosporium*	64	45	11	55	149	19	343

Data was searched and retrieved from the MycoCosm

### ABC transporters

In total, a versatile set of 49 genes encoding predicted ABC proteins were identified in the genome assembly of *P. radiata* (Table 6, Additional file 7). Out of them, 40 genes encode predicted ABC transporters, and 9 genes encode ABC proteins without any transmembrane domains. One of the 49 genes is apparently disrupted by a retrotransposon insertion (ABCF3 encoding gene) (Additional file 7). All types of ABC transporter encoding genes, which are commonly found in the species of *Basidiomycota* class *Agaricomycetes* could be identified [41, 42]. Not surprisingly, the set of putative ABC proteins of *P. radiata* demonstrates high similarity with the set of ABC proteins found in the genomes of *P. brevispora* [41] and *P. centrifuga* (this study). The differences between the three species of *Phlebia* are merely quantitative (Table 6), probably due to a few lineage-specific cases of gene loss or duplication events.

**Table 6:** Number of ABC transporter encoding genes identified in the genomes of the seven phlebioid fungi

Fungal species / ABC Subfamily	*P. radiata*	*P. brevispora*	*P. centrifuga*	*B. adusta*	*P. gigantea*	*P. carnosa*	*P. chrysosporium*
ABC-A	1	1	1	0	0	0	0
ABC-B (FL)	3	3	4	4	3	3	3
ABC-B (HT)	8	6	6	8	9	7	8
ABC-C	18	26	20	13	19	18	18
ABC-D	2	2	2	2	2	2	2
ABC-E	1	1	1	1	1	1	1
ABC-F	5	5	5	4	5	5	5
ABC-G	8	8	7	7	6	8	10
ABC-I	3	3	3	3	3	3	3

Data was searched and retrieved from the MycoCosm

Compared with the four other phlebioid clade species (*B. adusta*, *P. gigantea*, *P. carnosa*, *P. chrysosporium*), a few notable differences may be noticed (Additional file 8). The three species of *Phlebia* all possess one gene encoding a transporter of ABC-A family, whereas corresponding genes are missing from the other phlebioid species (Table 6). ABC-A transporters are likely involved in the transport of lipid molecules, and they were previously identified in most of the analyzed fungi of the *Basidiomycota* subphylum *Agaricomycotina* [41, 42]. Likewise, a soluble ABC protein classified as ABCF3 encoding gene was identified in the genomes of *P. brevispora*, *P. centrifuga* and *P. radiata* (Table 6, Additional file 8). The function of ABCF3 encoding gene is unknown, and it is apparently non-essential, since the gene is absent from ca. 40% of the analyzed species of *Agaricomycotina*. We could not identify the gene in the *Phanerochaete* clade, and in *P. radiata*, the corresponding gene is disrupted by an insertion of a retrotransposon, thereby most likely making it non-functional. Another gene classified as ABCG6.2 and encoding an ABC transporter of unknown function was found in *P. chrysosporium, P. carnosa* and *P. gigantea*, but not in the species of *Phlebia*. This gene, however, was previously identified in less than 25% of the analyzed species of *Basidiomycota* [41, 42]. Another notable feature of the set of ABC genes in *P. radiata* is a high number of ABCC4 encoding genes, present in eight copies in the most expanded subfamily of ABC-C proteins (Table 6, Additional file 7).

### Secondary metabolism genes of *P. radiata*

Bioinformatic analysis of secondary metabolite (SM) biosynthesis-related genes of *P. radiata* indicates that the genome harbors a rich repertoire of genes putatively involved in the production of secondary metabolites. At this stage, however, no accurate chemical structures of the potential products could be inferred (Additional file 4).

#### Polyketide synthases

Six genes encoding predicted polyketide synthases (PKSs) were identified in the genome assembly of *P. radiata* (Pks1-6) (Additional file 4). Four were annotated as type I PKS encoding genes (Pks1, Pks2, Pks4 and Pks5), whereas two genes are predicted to encode a type III PKS (Pks6) and a hybrid PKS-NRPS (non-ribosomal peptide synthase) (Pks3), respectively. Similar hybrid PKS-NRPS-encoding genes are found in a number of wood-inhabiting *Basidiomycota Agaricomycetes* fungi including *Heterobasidion irregulare* [43]. Based on the presence of conserved modules recognized in the deduced proteins, the identified type I PKSs could be further classified into one non-reducing PKS (Pks2) and three reducing PKSs. Among the three reducing type I PKS genes, Pks4 and Pks5 share 73 % of amino-acid sequence identity, suggestive of a gene duplication. Two of the identified PKS encoding genes form larger SM pathway gene clusters. The non-reducing type I PKS-encoding Pks2 gene was identified next to a NRPS-like encoding Nrl6 gene (see below for the discussion of this gene family) together with three genes encoding cytochrome P450 redox enzymes and one predicted gene coding for a glycosyl transferase. Interestingly, GH76 and GH16 CAZyme genes are located close to Pks2 gene. Pks4 was observed to be clustered with the NRPS-like gene Nrl8, in addition to two genes encoding MFS transporters and several genes encoding putative tailoring enzymes, indicative of functional roles in chemical modifications of the compounds produced by the corresponding PKS enzymes.

#### Adenylate-forming reductases

Members of this protein family resemble non-ribosomal peptide synthases (NRPS), but lack a condensation domain [44]. Although no canonical NRPS genes were identified in the genome of *P. radiata*, however, nine genes encoding predicted adenylate-forming reductases were found. Based on their domain organization, one gene (Lys2 homolog) was identified as a putative L-α-aminoadipate reductase, which is involved in L-lysine biosynthesis in fungi [44]. The remaining eight genes (Nrl1-8) encode adenylate-forming reductases of unknown function, and several of them are clustered with other SM encoding genes: Nrl1 is clustered with a predicted terpene cyclase, Nrl6 and Nrl8 are located close to the predicted PKS genes, Nrl7 is adjacent to a cytochrome P450 encoding gene, whereas the genes for Nrl2, Nrl3 and Nrl4 form a cluster on their own, with near location to the gene for Nrl5. Interestingly, a GH12 endoglucanase gene, putative AA3 alcohol oxidase and aryl-alcohol oxidase encoding genes, and a GH131 cellulase gene are found close to the cluster of Nrl2, Nrl3 and Nrl4. In addition, the gene for Nrl7 adenylate-forming reductase was recognized to be located next to an AA9 family LPMO encoding gene. Interestingly, expression of this AA9 gene was not up-regulated on spruce wood substrate [26] indicating a distinct function for the specific AA9 enzyme other than attack on the cellulose polymer. Within the gene clusters involved in SM biosynthesis, genes encoding various oxidases indicate their functional activity in modifications of the products of the SM pathways.

#### Terpene cyclases

Predicted terpene compound forming terpenoid cyclases (TC) [45] constitute the most numerous group of the identified SM biosynthesis involved genes in the genome of *P. radiata*. In total, 16 putative TC encoding genes (Ter1-Ter16) were identified, of which one (Ter16) could encode a squalene synthase. The products of remaining genes are unknown. Many of the TC genes are located in clusters: Ter5, Ter6 and Ter7 and Ter8 – Ter11 form two separate clusters on unitig70; Ter14 clusters with Ter15, and Ter1 is located next to the adenylate-forming reductase encoding gene Nrl1. There is a CAZyme gene cluster close to the cluster of Ter8-Ter11 including two GH5 genes and a putative AA3 family alcohol oxidase encoding gene. Ter2 gene is also located between two AA3 family genes and next to a cytochrome P450 gene.

#### AMP-dependent acyl-CoA synthetases

Our bioinformatics analysis identified two additional genes coding for unknown functions, which, based on their predicted protein domain organization, could have a role in secondary metabolism. Both genes share some similarity with AMP-dependent acyl-CoA synthetase encoding genes, and their deduced protein products enclose a NRPS-specific C-terminal domain. The function of these proteins is still obscure.

Among the multiple predicted proteins involved in biosynthesis of secondary metabolites in *P. radiata*, six genes coding for Nrl4, Pks6, Ter1, Ter3, Ter4 and Ter12 were earlier recognized as being up-regulated in the *P. radiata* transcriptomes on spruce wood substrate (at 2-week and 4-week time points) [26] (Additional file 4). This indicates specific functions and involvement of chemically diverse fungal secondary metabolite compounds in active hyphal growth and colonization of wood, and potential participation in the wood decomposition processes.

### Small secreted proteins of *P. radiata*

A total number of 956 genes identified in the *P. radiata* genome were predicted to encode secreted proteins, among which 215 represented small and cysteine-rich proteins (small secreted proteins, SSPs). From the *in silico* predicted protein secretome, 83 and 307 proteins were selected by EffectorP and LOCALIZER, respectively, as putative SSP candidates. Afterwards, all SSP candidates were merged and the annotated and predicted CAZymes were removed from the final analysis, thereby resulting in a final set of 430 candidate proteins as SSPs (Additional file 5).

**Fig. 7:**
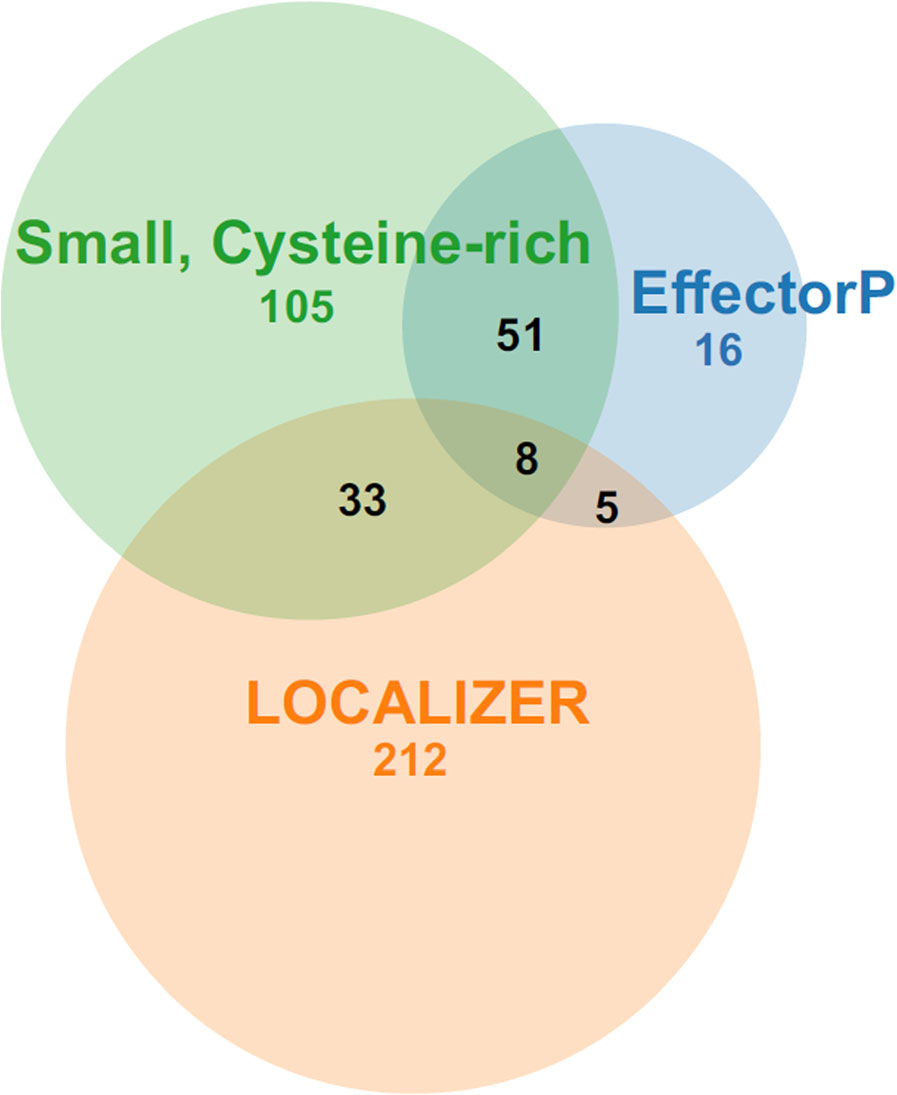
Venn diagram illustrating the number of shared SSP candidates of *P. radiata*. The SSP genes were computationally analysed by using EffectorP and LOCALIZER and according to the criteria of being small and cysteine-rich

The Venn diagram depicts the number of SSPs obtained according to the three criteria used (Fig. 7). Eight SSPs (genes minus.g2681, minus.g6180, plus.g1485, plus.g2909, plus.g2957, plus.g7370, plus.g8107, plus.g881) were shared by all criteria, and could be deemed as good SSP candidates for functional validation after gene structural inspection (Table 7). In the set of predicted SSPs in *P. radiata*, the shortest SSP candidate was only 66 amino-acids in length (gene plus.g1238.t1), while the longest protein was 1718 aa in length (gene plus.g13035.t1). The average length for the translated proteome, secretome and the selected SSPs was in descending order (487, 394 and 353 aa, Additional file 6), opposite to the average of cysteine composition in the protein models, which were 1.49 %, 2.12 % and 3.01 % for the three protein sets, respectively. According to the *P. radiata* transcriptome data [26] (Additional file 5), the top up-regulated SSP genes were found to encode putative hydrophobins. Hydrophobins are surface-active small secreted proteins that have diverse roles in fungal hyphal and mycelial growth and lifestyles [46].

**Table 7:** Summary of the SSP candidates of *P. radiata* that fulfilled the three criteria used in the analyses

Protein coding gene ID	Length	Number of cysteines	EffectorP^a^	LOCALIZER^b^
minus.g2681	265	10	0.610	NLS
minus.g6180	200	6	0.669	0.998, C
plus.g1485	119	11	0.916	NLS
plus.g2909	150	8	0.676	0.998, C
plus.g2957	146	8	0.570	0.896, C
plus.g7370	229	19	0.767	0.716, C
plus.g8107	158	8	0.747	0.971, C; 0.824, M
plus.g881	179	4	0.781	0.993, C; 0.995, M

^a^The number is the probability of being an effector given by EffectorP

^b^The number is the probability of having a Chloroplast (C) transit peptide-like or Mitochondria (M) transit peptide-like sequences given by LOCALIZER; for the cases of searching for nuclear localization signals (NLS), no probability was returned

## Discussion

*Basidiomycota* species of the phlebioid clade of the systematic order *Polyporales* [10, 11] are wood-inhabiting fungi with biotechnological interest due to their ability to decompose all components of wood lignocelluloses, including modification and degradation of wood lignin [5, 12, 26]. Phlebioid fungi include the model species of white rot decay of wood, *Phanerochaete chrysosporium* [12] together with the efficient lignin-attacking species *Phlebia radiata* [18, 26] and xenobiotics modifying species *Bjerkandera adusta* [7, 47], all of which are well-known producers of a large array of secreted enzymes against wood carbohydrates (CAZymes) including numerous auxiliary oxidoreductases (oxidases, laccases, peroxidases and monooxygenases) [5, 13, 33]. Especially, production of the oxidoreductases to generate extracellular ROS (reactive oxygen species) [48] and to modify lignin by production of lignin-active class-II peroxidases [13, 14, 37] are characteristics of the phlebioid species. The strong ability for production of ROS and oxidoreductases inherent among phlebioid fungi facilitates the attack on lignin and crystalline cellulose which furthermore allows access to the more easily degradable and utilizable plant biomass polysaccharides.

Of the phlebioid species selected for this comparative study, *P. chrysosporium* has been the most extensively studied and was the first genome of *Basidiomycota* fungus to be sequenced in early 2000 [12]. Since then, hundreds of *Basidiomycota* including over 80 species of the order *Polyporales* have been genome sequenced in the 1000 Fungal Genomes project (JGI MycoCosm https://genome.jgi.doe.gov/programs/fungi/index.jsf) [16, 49]. The sequenced and annotated phlebioid clade species of *Polyporales* comprise one or a few species of the genera *Phanerochaete*, *Phlebia*, *Phlebiopsis*, and *Bjerkandera*. Although the phlebioid fungi are deadwood and woody debris colonizing and decomposing species, they deviate in their ecological and physiological characteristics. *P. chrysosporium* has been mainly isolated from hardwoods (angiosperm wood) whereas *Phanerochaete carnosa* usually inhabits softwood (coniferous wood) [29]. *Phlebiopsis gigantea* is considered as a pioneer colonizer of softwood due to its special ability to grow into freshly exposed conifer sapwood, such as cut tree stumps, with a high content of resinous extractives, which apparently restricts substrate access for other wood-decay fungi [15]. *Bjerkandera adusta* in turn is a common *Polyporales* species in temperate European forests inhabiting dead angiosperm hardwood, such as beech, and coniferous wood. *B. adusta* is considered as a secondary or primary colonizer of attached branches, tree stumps and fallen wood [50].

*Phlebia* species, on the other hand, are likewise secondary or primary colonizers able to cause white rot both on coniferous and hardwood, with some substrate specificities [51, 52]. The resupinate fruiting bodies of *P. radiata* are commonly found on dead angiosperm wood such as stumps and branches of birch and rowan, whereas *P. centrifuga* is a common colonizer of dead conifers in the boreal forests of North Europe [51]. *P. brevispora* was described as a new species causing substantial white rot decay in pine wood in North America [52]. Thus, by the aid of comparative genomic analyses on the wood carbohydrate and lignin active CAZyme encoding genes, we expected to observe some differences in the gene repertoire between the seven species of taxonomically near-related and eco-physiologically specified phlebioid white-rot fungal species.

As an outcome of the complete sequencing, assembly, gene prediction and annotation of the *P. radiata* nuclear genome, a fully closed and gene dense reference genome was obtained. Most of the sequenced phlebioid genomes are draft assemblies and there has been a need for a more accurate reference genome, which will now be filled by the availability of *P. radiata* genome. The predicted gene number of *P. radiata* is the second highest among the phlebioid genomes. Specific features of the predicted protein-coding genes in *P. radiata* were the extremely short exons, even as short as two nucleotides, and many exons comprising only one codon (three nucleotides). For example, the glycolytic core metabolic GAPDH enzyme encoding gene begins with the first start codon exon followed by the immediate first intron (gene plus.g5457). In addition, the 5´ UTRs of the annotated genes of *P. radiata* typically contain putative upstream open reading frames (uORFs). In fungal mRNAs, uORFs are fairly common and are believed to have for example regulatory roles for gene expression and possibly an effect on mRNA stability [53].

Exploring the CAZyme gene content among the seven phlebioid fungi revealed gene sets typical for white rot *Basidomycota* fungi of the class *Agaricomycetes* [5, 7, 8, 13, 28, 30] encoding an array of secreted cellulases, hemicellulases, pectinases, auxiliary oxidoreductases and lignin-modifying enzymes. In addition to the conventional cellobiohydrolase encoding genes of the families GH6 (1 gene in phlebioid genomes) and GH7 (3-8 genes in each fungus), all the studied genomes harbor GH131 genes belonging to a novel CAZy family, which includes unusual hydrolases with bifunctional exo-β-1,3-/-1,6- and endo-β-1,4 activities towards a wide range of β-glucans including also cellulosic derivatives [54]. Another unconventional cellulase encoding gene present in the phlebioid genomes belongs to the family GH9. The *P. chrysosporium* GH9 gene has been cloned and characterized [55] and GH9 enzyme of the bacterium *Clostridium cellulosi* was recently described as a processive endoglucanase [56]. It is of interest that the brown rot fungal genomes of *Polyporales* seemingly lack GH9 genes [57]. Thus, it may be proposed that the higher degree of hydrolytic enzyme diversity against cellulose gives an advantage for the white rot phlebioid fungi for efficient decomposition of wood lignocellulose.

The CAZy family AA9 lytic polysaccharide monooxygenases attack and cleave polymeric cellulose via oxidative and oxygen atom incorporating mechanism [48, 58, 59] and may also act on hemicelluloses [60]. The AA9 *lpmo* genes are expanded in white rot *Polyporales* fungi [57], and accordingly, the studied phlebioid genomes contained over 10 annotated *lpmo* AA9 genes. Especially notable is the large number of AA9 family genes (up to 28 genes) in *B. adusta*. The novel auxiliary enzyme LPMO family AA14 encoding genes were additionally identified in the seven phlebioid genomes, indicative of enzyme activities necessary for efficient white rot fungal lifestyle, although the number of genes (2-3) was much lower than annotated for the AA9 family LPMOs. The family AA14 LPMOs were recently discovered to present oxidative reaction against the heteroxylan shield covering cellulose microfibrils [35], thus potentially facilitating access to cellulose backbone for the cellulolytic enzymes.

Taking into account the recent finding of activation of LPMOs preferentially by hydrogen peroxide (rather than dioxygen) [48, 61], and the requirement of hydrogen peroxide as the primary oxidant for the CAZy family AA2 lignin-attacking class-II peroxidases [5, 37], enzymatic production of H_2_O_2_ and other ROS for biological decomposition of lignocellulose is of utmost importance. Of the various auxiliary oxidoreductase enzymes, the potential hydrogen peroxide producing GMC flavoprotein family AA3 is presented with 23-39 genes in the phlebioid fungi, and all the studied genomes harbor one *cdh* gene encoding a cellobiose dehydrogenase (CDH, AA3_1). The specific function of the hybrid flavo-hemoprotein CDH is still unclear; in addition to possibly participating in lignin conversion, the enzyme may be involved in attack on cellulose and hemicellulose in cooperation with LPMOs [62, 63].

A wide range of activities are needed for the degradation of the complex hemicellulose polymers [64, 65]. Endo-xylanases and mannanases cut the polymeric xylan and mannan backbone, respectively, whereas mannosidases and xylosidases release monomeric sugars. For example, acetyl xylan esterases, acetyl esterases, glucuronyl esterases, glucuronidases, galactosidases, fucosidases and arabinofuranosidases are needed for releasing the various linkages and side chains moieties present in chemically and structurally diverse hemicelluloses [64]. Genes encoding all activities necessary for hemicellulose decomposition were detected in the studied phlebioid genomes, although some differences in the set of genes were observed. For example, the CAZy family GH29 α-fucosidase encoding genes were present only in the *Phlebia* species, and interestingly, these enzymes could be important in the early phases of wood colonization [9, 43]. Furthermore, the family GH11 endoxylanases may imply specific functions due to their uneven distribution as corresponding genes among the phlebioid genomes. In the case of *P. gigantea*, the lack of GH95 α-fucosidase encoding genes (1-3 genes in other phlebioid genomes) as well as the abundance of CE8 pectin methylesterase encoding genes were noticed in the previous genome description study [15].

The phlebioid genomes possess key genes encoding activities against lignin including genes of AA2 family class II peroxidases together with genes of AA3, AA4, AA5 families encoding proteins for production of hydrogen peroxide. The genera *Phlebia*, *Phanerochaete* and *Bjerkandera* present various types of MnP and LiP enzymes, including one VP in *B. adusta* [14], whereas *Phlebiopsis gigantea* genome is restricted to short- and atypical MnPs [15]. It is evident that the class-II heme peroxidases are necessary for oxidation and modification of lignin [37], and the appearance of the first class-II peroxidase gene in the ancestor of *Agricomycetes* 290 million years ago initiated decomposition of wood lignin and white rot lifestyle [13].

Notably, expansion in the number of AA2 class-II peroxidase encoding genes (10-21 genes per genome) were annotated with several enzymes produced and secreted on wood substrates in the most efficient white rot colonizers and lignin-degrading fungi (*Phanerochaete chrysosporium, P. carnosa, Phlebia radiata, Bjerkandera adusta*) [12, 13, 26, 29, 57]. The expansion of AA2 lignin-modifying peroxidase and dye-decolorizing peroxidase (DyP) to 21 and 10 genes, respectively, in *B. adusta* was previously documented [14]. The evolutionary analysis suggested that the ancestor of *Agaricomycetes* possessed one to two genes for class-II peroxidases, and one to two genes coding for dye-decolorizing peroxidases [13]. The number of DyP encoding genes in the phlebioid fungi has expanded from one to four genes in *P. gigantea* and to ten genes in *B. adusta*, whereas the efficiently lignin-attacking species *P. chrysosporium* has evidently lost this gene. Thus, it may be concluded that on the contrary to the class-II LiP, MnP and VP peroxidases, DyP enzymes are not essential for oxidization and degradation of lignin.

Overall, the CAZyme gene content of the studied fungal genomes was quite constant, with over 150 annotated genes per genome, which is expected from closely related fungi with similar, white-rot decay and wood inhabiting lifestyles. However, some fine-tuning of plant cell wall degradation activities may be proposed from the subtle differences detected, especially in the number of CAZyme genes coding for enzyme activities against hemicelluloses. In addition, the family GH44 endoglucanase encoding genes were found to be specific for the three *Phlebia* species. Moreover, genomic clustering of especially AA9 *lpmo* genes and GH10 xylanase encoding genes was noticed to be common among the phlebioid fungi, which may imply concerted regulation of gene expression [66].

Phylogenetic conservation of specific gene clusters pinpoints their importance in the fitness for fungi in their environments, and thereby, preservation in the course of evolution [67]. Clustering of lignin-modifying peroxidase and copper radical oxidase genes of *P. chrysosporium* has been recognized previously [12], whereas clustering of cellulase encoding genes was less evident. Similarly, clustering of the *P. carnosa* MnP and LiP encoding genes has been observed, whereas most of the CAZyme genes were loosely associated [29]. In *P. radiata*, we noticed in this study similar features of clustering of LiP encoding genes (two genes) and likewise noticed in *P. chrysosporium*, together with AA5 copper radical oxidase encoding genes (three genes). For gene clustering and co-expression, several explanations may be proposed. First, the neighboring genes may locate in the genome on the same euchromatin or heterochromatin segment, or the co-expressed genes may contain a common transcription factor binding site (on the promoter region) or even share the same promoter [66]. However, as clustering of genes is not necessary for co-expression, the true purpose of gene clustering may be positioning the physiologically important and beneficial genes into chromosomal areas of low recombination rate in the fungal genomes [38, 67].

A holistic study of the transcriptome and proteome of *P. radiata* was recently conducted, which confirmed that all proteins necessary for the white-rot type of decay are produced on solid spruce wood, and their corresponding genes were up-regulated [26]. The time scale analysis of proteomics revealed that several lignin-modifying class-II peroxidases together with glyoxal and alcohol oxidases were abundantly produced at earlier growth stage on wood, indicating an initial oxidative attack against lignin units. Dynamic changes in quantities of especially LiP and MnP enzymes were detected during the six-week cultivation on wood. *P. radiata* is also able to activate its wood decomposing enzymatic machinery under oxygen-limited cultivation atmosphere in order to bioconvert lignocellulosic waste materials while simultaneously producing ethanol and other metabolites [68, 69].

Of the multitude of protein-degradative activity (peptidase) encoding genes recognized in *P. radiata* genome, secreted peptidases produced during the six-week cultivation on spruce wood were previously identified in the proteome [26], and the abundance of peptidases was shown to increase over time. Together with an increase in the abundance of peptidases in the proteome on wood, the simultaneous increase of e.g. chitinases [26] indicated that the fungal cell wall is undergoing reorganization and essential nutrients, predictably mainly nitrogen compounds, were recycled upon growth on wood. The most abundant peptidases detected belong to the A01A and T01A subfamilies according to the MEROPS classification. In this study, annotation of the peptidase encoding genes revealed that *P. radiata* genome encodes 31 and 11 putative members of the peptidase A01A and T01A families, respectively, which explains their dominance in the expressed proteome on wood. The most abundant peptidases identified from the proteome included a M28 metalloprotease, an A01 aspartyl peptidase and an S53 tripeptidyl peptidase [26].

The diversity of genes potentially involved in biosynthesis of secondary metabolites (SM) observed in *P. radiata* is quite uncommon for a white rot *Polyporales* and class *Agaricomycetes* fungal species [8, 15], which is an indication of the ability of *P. radiata* to produce a diverse set of secondary metabolites. Altogether, genes for polyketide synthase, adenylate-forming reductase, terpene cyclase, and putative AMP-dependent acyl-CoA synthetase were identified and annotated in the genome of *P. radiata*. Protein family of adenylate-forming reductases is present in the *Basidiomycota* species, but the functions of these enzymes in fungi are not entirely understood [70]. Clustering of the secondary metabolism genes is commonly observed in fungi, which may contribute to the regulation of genes belonging to a single SM pathway [71]. The occurrence of secondary metabolism genes close to CAZyme genes, which was observed in *P. radiata* for a few occasions, has likewise been noted in the *Ascomycota* species *Trichoderma reesei* although the possible biological reason for this coincidence is not yet known [38, 72].

Effectors secreted by fungal plant pathogens during colonization of the host are well known examples of small secreted proteins (SSPs) [73, 74]. However, the role of SSPs in the lifestyle of saprotrophic fungi such as in the seven wood decay phlebioid species of this study, is still enigmatic. A recent study suggests that some of the SSPs identified in *Basidiomycota* genomes could participate in the lignin-attacking system [75]. Other possible roles for SSPs are development of multicellular structures, and coping with toxic compounds present in the environment [76] or perhaps, involvement in the interspecific fungal interactions. For example, one of the *P. radiata* genes predicted to encode a SSP (plus.g7370) is homologous with the *priA* gene *Lentinula edodes* for which a role during the early stage of fruiting body formation has been proposed [77].

One intriguing notion previously was the high expression level of hydrophobin-coding genes in *P. radiata* during colonization of spruce wood [26]. Hydrophobins are a large family of secreted proteins unique to filamentous fungi [78]. These proteins function by forming into polymeric, water-repellent monolayers at the surface of aerial structure such as spores, hyphae and fruiting bodies [78]. The surface (rodlet) layer coating the conidial spores of the pathogenic *Ascomycota* filamentous fungal species *Aspergillus fumigatus* was found to mask their recognition by mammalian immune system, thereby rendering silencing of immune response [79]. In the rice blast *Ascomycota* fungus *Magnaporthe grisea*, hydrophobins have been considered as pathogenicity factors involved in conidial development and viability, appressorium formation, infectious growth in host cells and possibly in cell wall assembly [80]. Our data could provide an example of more roles of hydrophobins in the *Basidiomycota* and in general, in saprotrophic fungi.

Analysis of putative ABC transporters indicated that the set of *P. radiata* ABC genes identified in the genome is characteristic for the species of the *Basidiomycota* subphylum *Agaricomycotina* [41, 42]. A notable feature of the set of ABC genes in *P. radiata* is a high number of genes coding ABCC4 transporters, particularly present in eight copies in the most expanded ABC-C subfamily, but all genes and their protein products of yet unknown function. Previous phylogenetic analysis indicates that numerous lineage-specific duplication events of this gene occurred in the evolution of *Agaricomycotina* [41], but their functional significance remains obscure. The subfamily of ABC-C transporters of unknown function, are depicted in great variation in copy number among *Agaricomycotina*. The gene is present in a single copy in about one third of the analyzed species, but in some species, in particular in the members of *Polyporales*, more than 10 genes are recognized [41], which furthermore implies the necessity of a versatile set of transporter proteins for saprotrophic lifestyle wood-decay fungi to enable efficient nutrient uptake and metabolic activity in their harsh habitats.

In conclusion, it may be inferred that the genome of *Phlebia radiata* undoubtedly places the fungus and species among the phlebioid clade of *Polyporales*, class *Agaricomycetes*, within the fungal phylum *Basidiomycota*. Especially the comparative genomic analyses of the sets of CAZyme and auxiliary oxidoreductase enzyme encoding genes among the seven phlebioid fungal genomes emphasize the significance of the wide array of genes for activities against all polymeric components of plant cell wall (cellulose, hemicelluloses, pectin, lignin) for a saprotrophic, white rot type of wood decay strategy, and successful lifestyle in the forest environments.

Importantly, many CAZyme genes were clustered in the genome, and interestingly, some CAZyme genes show near location with secondary metabolism biosynthesis genes, which suggests potential co-expression and functional roles for the produced metabolites in inhabitation and decomposition of wood by *P. radiata*. New findings on the abundance and versatility of the ABC transporter and small secreted protein encoding genes will aid in identifying them more extensively in the fungal genomes and transcriptomes. Our comparative approach and bioinformatic results are adding up the fundamental knowledge on fungal genomics and biology, which may be employed in research on fungal metabolic processes and bioconversions, as well as a source for new genes and proteins for biotechnology applications.

## Methods

### Fungal strain and isolation of DNA

*Phlebia radiata* Fr. isolate 79 (FBCC0043) was originally isolated from a decaying grey alder (*Alnus incana*) trunk in South Finland, and is deposited in the Microbial Domain Biological Resource Centre HAMBI (HAMBI-FBCC subcollection, kotka.luomus.fi/culture/fbcc) of the University of Helsinki. Identification of the isolate has been verified by ITS-PCR [10, 81]. The isolate was cultivated and maintained on malt extract (2 % w/v) agar at 25 °C and in the dark throughout the study. For isolation of total DNA, *P. radiata* was cultivated in liquid malt extract (2 % w/v) medium for 7-14 days at 25 °C in the dark after which the mycelium was harvested, frozen to -20 °C and lyophilized. Isolation of genomic DNA was initiated with CTAB-supplemented incubation of fine ground mycelium followed by phenol-chloroform purification, RNAse treatment, and final precipitation in pure cold ethanol [82]**.**

### Genome sequencing, assembly and gene prediction

Prior to sequencing, purity and molecular size distribution of total DNA (2.0 mg) was analysed by Qubit (Thermo Scientific) and Bioanalyzer (Agilent Technologies). PacBio single molecule, real-time (SMRT) sequencing technology (Pacific Biosciences of California) was the basic method used to obtain long nucleotide reads for *de novo* assembly of the genome. In total, 9 SMRT cells were run which yielded 70 2780 reads with N50 read length of 9 384 bp and a total of 4.29 Gbases of nucleotide sequence data**.** HGAP3 from SMRT Analysis v2.3 suite was used with 6 000 bp seed read length to assemble the genome. The assembly was polished with three subsequent runs of SMRT Analysis re-sequencing pipeline with Quiver consensus algorithm. Low coverage (<5X) and redundant, such as ribosomal only, short contigs were removed. Strand-specific paired-end Illumina RNA-Seq data described in [26] was mapped against the final assembly of genome sequence using the splice-aware RNA-Seq aligner STAR [83]. Mapped RNASeq data was filtered using the following parameters: 1) both R1 and R2 were properly mapped, 2) insert size between the reads was more than 500 bp, and 3) the mapping quality was 255. The cleaned RNA-Seq bam file was finally divided into plus and minus strand read pairs based on the strand-specific nature of the libraries (R2 data corresponding the gene transcription orientation) before genome annotation. Coding sequences of protein-coding gene models were predicted by the BRAKER1 software [84] using the plus and minus strand RNA-Seq evidence separately, and functionally annotated by blastp (version 2.2.30) [85] searches against the NCBI non-redundant protein sequences database, by PANNZER [86] and by Blast2GO [87] software. Gene models of interest were manually curated.

### GO enrichment analysis

For functional prediction, GO enrichment analysis based on Fisher´s exact test was conducted by using Blast2GO [87] with data from our previous transcriptome study [26]. Filter mode was p-value of 0.05 for statistical significance. Genes up-regulated (p-value < 0.05, log2 FC ≥ 1) upon growth on spruce wood as compared with malt extract medium as substrate at the two-week and/or four-week time points of cultivation were selected for the analysis.

### Annotation and comparison of CAZyme and peptidase encoding genes

*P. radiata* CAZyme genes were functionally annotated previously [26]. Majority of the CAZyme encoding genes involved in decomposition of plant cell wall lignocellulose were manually curated. Information on annotation of putative *P. radiata* peptidases according to the MEROPS peptidase database [40] (https://www.ebi.ac.uk/merops/) was collected from the Joint Genome Institute (JGI) MycoCosm fungal genomics repository [16] (https://genome.jgi.doe.gov/programs/fungi/index.jsf). For comparative genomics of the CAZyme and peptidase genes, genetic information of the phlebioid fungi *Phlebia brevispora* (HHB-7030 v1.0), *Phlebia centrifuga* (FBCC195), *Phlebiopsis gigantea* (11061_1 CR5-6 v1.0), *Bjerkandera adusta* (HHB-12826-SP v1.0), *Phanerochaete chrysosporium* (RP-78 v2.2) and *Phanerochaete carnosa* (HHB-10118-Sp v1.0) was downloaded from the JGI MycoCosm.

### Genes for secondary metabolism and ABC transporters

Identification of the secondary metabolism-related genes in *P. radiata* genome was performed using antiSMASH prediction platform [88] with the default settings for fungal genomes followed by manual curation and annotation of the identified genes. *P. radiata* ABC protein-coding genes were identified by multiple tblastn and blastp searches against the genome assembly and a set of predicted proteins, respectively. Sequences of previously identified *P. brevispora* ABC proteins [41] were used as queries. All hits producing E-values below 10^-6^ were further examined. Gene models were manually curated and, when necessary, the positions of N and C termini were adjusted. For gene number comparisons, the number of ABC transporter genes of the studied phlebioid fungi was obtained from the previous study [41]. The putative ABC transporter genes of *P. centrifuga* were then identified by blastp searches against the genome sequence deposited in the MycoCosm with the *P. radiata* ABC proteins as queries.

### Small secreted proteins

Secreted proteins were identified using SignalP v.4.1 (sensitive mode) [89], TargetP v.1.1 [90] and TMHMM v.2.0 [91] to predict the presence of signal peptide, targeted cellular localization and transmembrane domain (TM), respectively. Proteins having more than two TMs and or having a single TM not overlapping with the signal peptide (outside the first 60 amino acids) were excluded. Subsequently, the predicted secretome was submitted to EffectorP v.2.0 [92] and LOCALIZER v.1.0 [93]. EffectorP could help prioritize the selection of highly confident effector candidates. LOCALIZER aids the prediction of translocation of fungal effectors into plant chloroplasts, mitochondria and nuclei based on the presence of transit peptides and nuclear localization signals. In addition, secreted proteins with length < 300 amino acids and cysteine residues ≥ 4 were also selected. All three sets of putative proteins were merged and constituted the final SSP list after removing the putative secreted CAZymes.

### Recognition of genomic clustering and co-expression of CAZyme genes

Co-localization of *P. radiata* CAZyme genes in the genome assembly were searched according to unitig number and nucleotide start and end positions of the predicted gene models. Minimum of three CAZyme genes in a window of maximum of 11 genes was considered as a cluster. Size of the window was calculated according to [38]. Common expression patterns i.e. up-regulation at 2 and 4 weeks of growth on spruce wood substrate as compared with malt extract liquid medium were identified [26]. Co-localization of the GH10 and AA9 family genes of *P. brevispora*, *P. centrifuga*, *P. gigantea*, *B. adusta*, *P. chrysosporium* and *P. carnosa* was investigated with the graphical display of the JGI Genome Browser (https://genome.jgi.doe.gov/help/browser_main.jsf) by visualizing the clusters of the CAZyme gene models positioned along each genome assembly scaffold. Maximum number of three non-CAZyme genes was allowed between the genes forming the cluster.

## Additional files

Additional file 1: GO enrichment analysis of *P. radiata* genes up-regulated in the presence of spruce wood. Statistically significant up-regulation (p-value < 0.05, log2 FC ≥ 1) was analyzed previously [26] as compared with malt extract cultivation at the two week (2w up-regulated) and/or four week (4w up-regulated) time points of cultivation. (XLSX 11 kb)

Additional file 2: *P. radiata* gene models encoding CAZymes, their locations in the genome assembly together with transcriptome data and proteome data from spruce wood cultivations [26]. 2w, two week time point of the spruce wood cultivation; 4w, four week time point of the spruce wood cultivation; up, up-regulated; down, down-regulated. (XLSX 44 kb)

Additional file 3: *P. radiata* gene models encoding plant cell wall attacking CAZymes and located in clusters in the genome assembly. Fold change of expression of the genes in spruce wood cultivations as compared with malt extract cultivations at the two week (2w) and four week (4w) time points of cultivation was analyzed previously [26]. (XLSX 22 kb)

Additional file 4: Putative secondary metabolism genes identified from the *P. radiata* genome assembly. Fold change of expression of the genes in spruce wood cultivations as compared with malt extract cultivations at the two week (2w) and four week (4w) time points of cultivation was analyzed previously [26]. (XLSX 15 kb)

Additional file 5: Putative small secreted proteins of *P. radiata* chosen according to EffectorP analysis, LOCALIZER analysis and by identifying small cysteine rich protein. Fold change of expression of the genes in spruce wood cultivations as compared with malt extract cultivations at the two week (2w) and four week (4w) time points of cultivation was analyzed previously [26]. (XLSX 40 kb)

Additional file 6: Distribution of length of *Phlebia radiata* whole proteome, secretome and SSPs. Numbers on the bar denote the average length of each set of protein sequences. (PDF 19 kb)

Additional file 7: ABC transporter genes identified from the *P. radiata* genome assembly. (XLSX 117 kb)

Additional file 8: Comparisons of the numbers of ABC transporter genes between different species of *Agaricomycotina*. (XLSX 23 kb)

## Data Availability

The annotated *P. radiata* genome assembly is available at the Joint Genome Institute (JGI) MycoCosm repository (https://genome.jgi.doe.gov/Phlrad1/Phlrad1.home.html). All data generated and analysed in the study are included in this published article and its supplementary information files.

## Abbreviations

AA: Auxiliary activity redox enzyme
CAZyme: Carbohydrate-active enzyme
CE: Carbohydrate esterase
GH: Glycoside hydrolase
LiP: Lignin peroxidase
LPMO: Lytic polysaccharide monooxygenase
MnP: Manganese peroxidase
NRPS: Non-ribosomal peptide synthase
nt: Nucleotide
PKS: Polyketide synthase
PL: Polysaccharide lyase
SM: Secondary metabolism
