# Supplementary figures and images for: Genome description of Phlebia radiata 79 with comparative genomics analysis on lignocellulose decomposition machinery of phlebioid fungi
Source: BMC Genomics. 2019 May 28;20:430. doi: 10.1186/s12864-019-5817-8 (PMC6540522; doi:10.1186/s12864-019-5817-8)

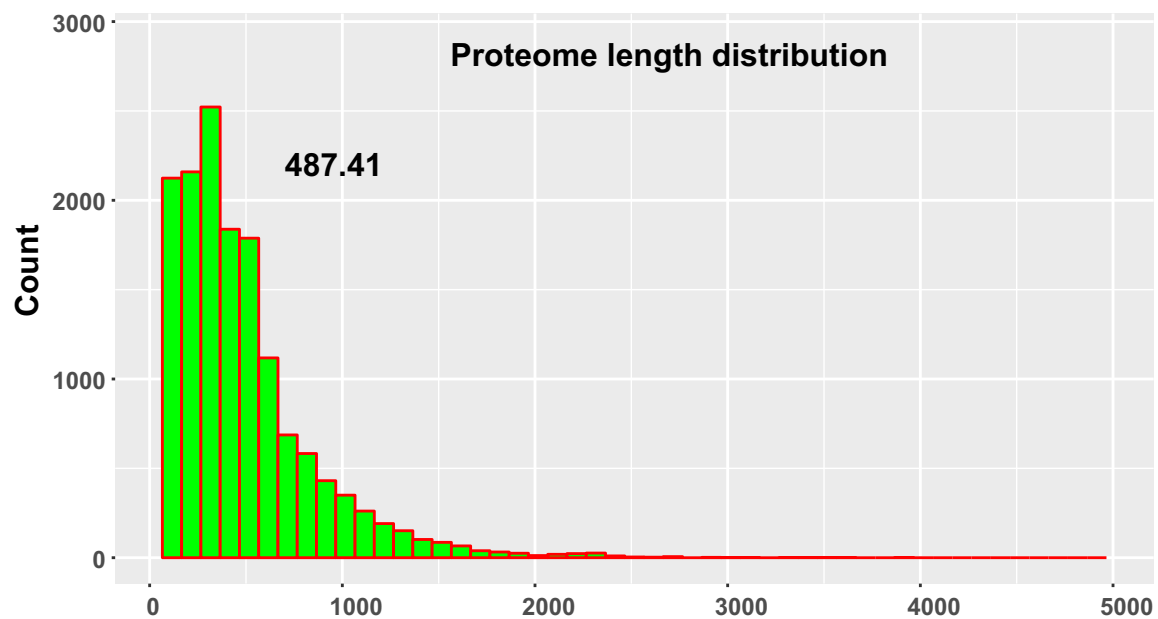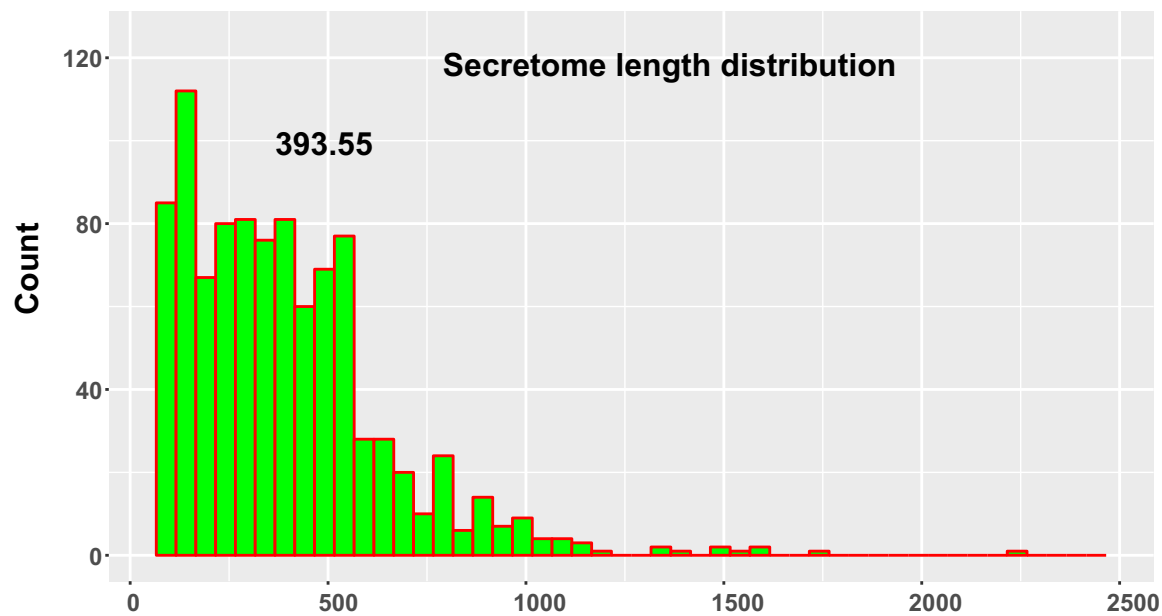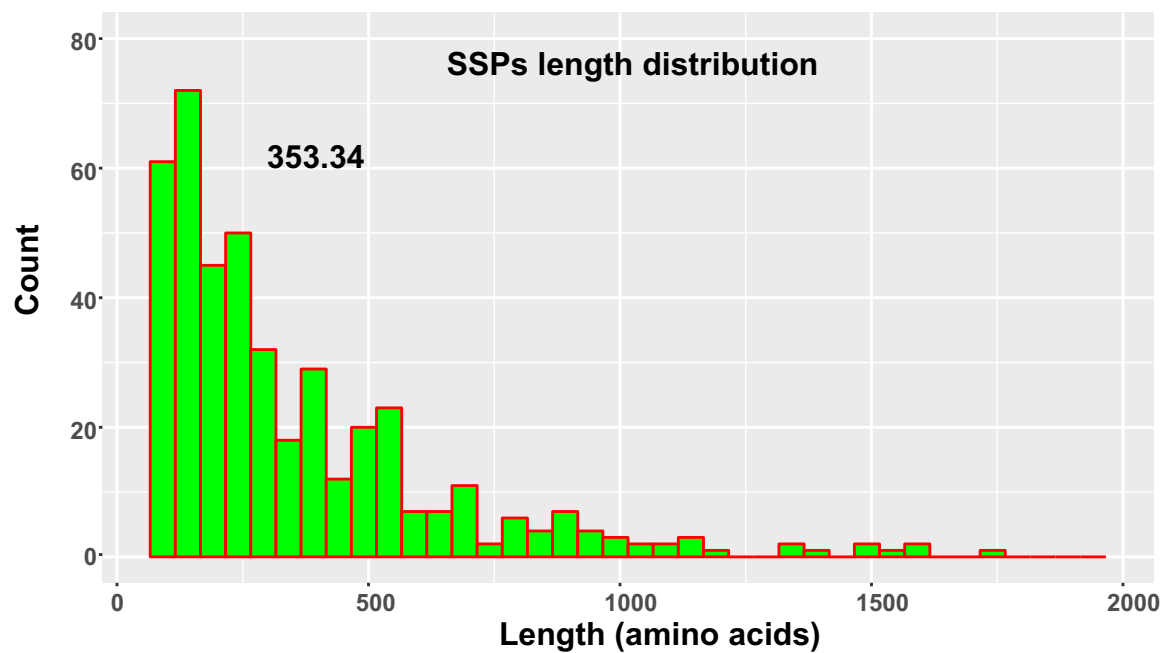

Supplement: Supplementary file 6 — Distribution of length of Phlebia radiata whole proteome, secretome and SSPs. Numbers on the bar denote the average length of each set of protein sequences. (PDF 19 kb) [file 12864_2019_5817_MOESM6_ESM.pdf]
